# Enhancing the Understanding of Resilience in Health Systems of Low- and Middle-Income Countries: A Qualitative Evidence Synthesis

**DOI:** 10.34172/ijhpm.2020.261

**Published:** 2021-01-16

**Authors:** Pauline Yongeun Grimm, Sandy Oliver, Sonja Merten, Wai Wai Han, Kaspar Wyss

**Affiliations:** ^1^Swiss Tropical and Public Health Institute, Basel, Switzerland.; ^2^University of Basel, Basel, Switzerland.; ^3^Social Science Research Unit, University College London, London, UK.; ^4^Faculty of Humanities, University of Johannesburg, Johannesburg, South Africa.; ^5^Department of Medical Research, Ministry of Health and Sports, Yangon, Myanmar.

**Keywords:** Health System Resilience, Health System Shocks, Qualitative Synthesis, International Health Regulations

## Abstract

**Background:** A country’s health system faces pressure when hit by an unexpected shock, such as what we observe in the midst of the coronavirus disease 2019 (COVID-19) pandemic. The concept of resilience is highly relevant in this context and is a prerequisite for a health system capable of withstanding future shocks. By exploring how the key dimensions of the resilient health system framework are applied, the present systematic review synthesizes the vital features of resilient health systems in low- and middle-income countries. The aim of this review is to ascertain the relevance of health system resilience in the context of a major shock, through better understanding its dimensions, uses and implications.

**Methods:** The review uses the best-fit framework synthesis approach. An *a priori* conceptual framework was selected and a coding framework created. A systematic search identified 4284 unique citations from electronic databases and reports by non-governmental organisations, 12 of which met the inclusion criteria. Data were extracted and coded against the pre-existing themes. Themes outside of the *a priori* framework were collated to form a refined list of themes. Then, all twelve studies were revisited using the new list of themes in the context of each study.

**Results:** Ten themes were generated from the analysis. Five confirmed the *a priori* conceptual framework that capture the dynamic attributes of a resilient system. Five new themes were identified as foundational for achieving resilience: realigned relationships, foresight and motivation as drivers, and emergency preparedness and change management as organisational mechanisms.

**Conclusion:** The refined conceptual model shows how the themes inter-connect. The foundations of resilience appear to be critical especially in resource-constrained settings to unlock the dynamic attributes of resilience. This review prompts countries to consider building the foundations of resilience described here as a priority to better prepare for future shocks.

## Background

###  Emergence of “Resilience” as a Concept in the Health Sector

 A country’s health system faces severe pressure when hit by an unexpected shock. The Ebola outbreak in West Africa that began in December 2013 took nearly a year until it was officially declared as an international public health emergency^1^; yet, by that time it had already spread to neighbouring countries and caused major disruptions in the functioning of their health systems and beyond.^1^ In 2018, the Ebola virus returned to the Democratic Republic of Congo. Despite the joint efforts of the Ministry of Health and relevant partners in implementing control measures, treatment and prevention efforts have been severely hampered by the on-going military conflict and civilian displacement.^2^ The concept of resilience has become ever more relevant with the current responses to the coronavirus disease 2019 (COVID-19) pandemic and is emerging as a prerequisite to building a strong health system that would withstand future health shocks.

 The term resilience was first used academically in the fields of mathematics and engineering^3,4^ and the key feature of resilience as that of ‘bouncing back’ to its original equilibrium after a displacement is known as *resilience engineering*.^5^ Resilience has a long history of application in art, literature, law, science, and engineering.^6^ This concept was soon after applied in the domains of ecology,^4^ and later to psychology where its early work focused mainly on trauma-affected individuals,^7^ and then to embrace the whole communities.^8^ Discourses on livelihood approaches have highlighted the social determinants of health by building resilience to improve healthcare access in the context of a wider society,^9^ which became the starting point of resilience-related research in the health system. The direct emergence of resilience in health, however, has been a relatively recent phenomenon, as a response to the Ebola crisis^10^ and there have been efforts to characterise and measure health system resilience to allow comparison across different contexts.^11^

 Health system resilience is defined as the capacity to prepare for and effectively respond to crises whilst retaining core health system functions when a crisis hits. Another aspect concerns the capability of the system to reorganise itself to meet the evolving needs of the situation.^10^

 It is paramount to note that the concept of resilience is not only applicable to acute external shocks but also to chronic conditions that have the potential to undermine the fabric of the health system. Examples are the organisational challenges resulting in human resource and drug shortages, which requires *everyday resilience*.^11,12^ Hence, building *everyday resilience* of the health system is linked with the strengthening of the overall health system.

 Several scholars among many embedded in health system’s research proposed conceptual frameworks in order to elucidate the complex nature of health system resilience. Blanchet et al developed a model of health system resilience, describing it as a governance capacity to absorb, adapt and transform itself in case of a shock.^13^ Kruk et al responded to demands from multilateral organisations in the aftermath of the Ebola crisis in 2014 by creating a framework describing five key characteristics of a resilient health system and a proposed resilience index corresponding to those five characteristics to measure resilience.^11^

###  Why Is This Synthesis Important?

 Despite a growing consensus that health system’s resilience is imperative in order to prepare for the next pandemic, a recent literature review revealed that there is a considerable gap between the theoretical foundations of resilience and its empirical application in the literature.^14^ Furthermore, due to the dearth of empirical evidence from low- and middle-income countries, there is little evidence to guide countries in strengthening health system resilience with contextualized application points for resource-constrained settings.

**Figure 1:**
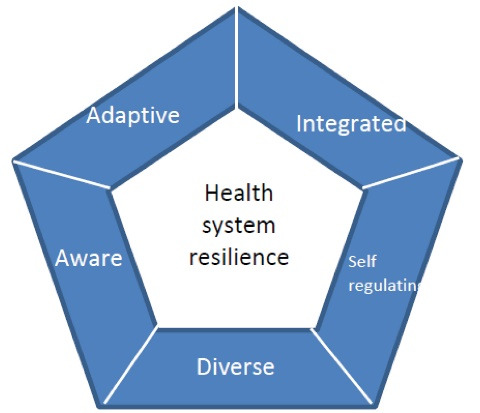
*A Priori* Framework for Health System Resilience. Source: Figure modified from Kruk and colleagues’ framework.^11^

 By exploring how the key five dimensions of resilient health system framework are applied in real life cases (Figure 1),^10^ the present systematic review provides a synthesis of the vital features of resilient health systems in low- and middle-income countries. The focus is on applied evidence of health system resilience using Kruk and colleagues’ health system resilience framework as the *a priori *conceptual framework.^11^

 As reiterated by various authors,^10,15-17^ the Ebola crisis in 2014 had been the defining moment upon which the concept of health system resilience, or the lack thereof, was brought to the domain of the health sector, and the international community has formed a consensus around the importance of building a foundation of resilient health systems. Therefore, only literature since the Ebola crisis in West Africa has been considered in our analysis. This review has also not included COVID-19 related studies as the search and analysis have been conducted before the occurrence of the pandemic. The findings and implications of this study, however, remain highly relevant, as low- and middle-income countries will be facing the devastating effect of the pandemic as we speak. Nevertheless, this has not been our scope and will require further research.

## Objectives

 The aim of this systematic review is to ascertain the relevance of the concept of resilience in relation to health systems in the context of a major shock, through a better understanding of its dimensions, uses and implications, as described in current literature. The framework synthesis will serve to improve understanding of:

1. the common key features of a resilient health system in low- and middle-income countries, building on Kruk and colleagues’ framework on health system resilience.
2. other features that are critical in the context of low- and middle-income countriesoutside of Kruk and colleagues’ framework that may indicate capacity for resilience.

## Methods

###  A Priori Conceptual Framework: Resilient Health System Framework 

 A best-fit framework synthesis approach was selected for this qualitative synthesis, as this method provides a means to build on an existing published model and to test its relevance for a wider population.^19^ An *a priori* conceptual framework was first selected and a coding framework created based on its key themes and sub-themes. The selected *a priori* framework developed by Kruk et al was chosen due to its corresponding resilient index, as the authors deemed the measures relatively comparable and easily applicable across different contexts. In addition, Kruk and colleagues’ framework has already been empirically tested in the cases of Liberia’s Ebola epidemic, which provided a good rational for selection.^20,21^ The *a priori* framework is predicated on five essential characteristics that indicate a resilient health system.^10,11^

 The following coding framework was generated based on the *a priori* conceptual framework, which guided the subsequent data extraction and analysis processes. Other recurrent themes associated with resilience were also noted (Table 1).

**Table 1:** The Coding Framework: *A Priori *Themes and Sub-themes

**Themes**	**1. Aware**	**2. Diverse**	**3. Self-regulating**	**4. Integrated**	**5. Adaptive**
**Sub-themes**	(i) knows health system capacity	(i) effectively respond to range of health needs	(i) isolate threat and maintain core function	(i) coordinate with non-health actors	(i) shift resources to meet need
	(ii) knows risks and population	(ii) adequately finance health systems	(ii) leverage outside capacity	(ii) engage citizens and communities to build trust	(ii) promote rapid local decision-making
	(iii) communicate			(iii) link healthcare provision to public health	(iii) evaluate to improve
				(iv) coordinate primary and referral care	

####  Resilience and the Building Blocks of the Health System

 The themes and subthemes of Kruk and colleagues’ resilience framework and other recurrent themes were recognised as elements of resilience, matched to the six building blocks of the health system^22^ and noted as either a strength or a weakness in each study of health system responding to a shock. The elements that did not match any of the traditional building blocks were grouped under ‘others.’ The map was reviewed by all the authors and consensus was reached for it to be used as an analytical tool. The top five most prominent elements of the health system in the synthesis of all papers were later presented in the refined conceptual model of health system resilience as ‘fundamental elements of health systems.’

###  Search Strategy and Study Selection

 Literature search was conducted in September 2019. The following relevant electronic databases were searched for eligible studies: MEDLINE, Ovid [2014 onwards], Embase, Ovid [2014 onwards], Web of Science Core Collection, Scopus, Global Health Library for World Health Organization (WHO) databases (Regional indexes), Epistemonikos, and Evidence Aid.

 A filter was applied for qualitative studies published from January 2014 onwards, as this was the time when the concept of health system resilience first received attention after the Ebola crisis in Africa.

 We used supplementary search techniques to identify studies not indexed in the databases listed above, by searching grey literature. As the topic is at the nexus of public health, political science, social science, and international development, databases and websites pertaining to a range of these multidisciplinary topics were considered. The following sites were consulted:

- “Grey Matters”: https://www.cadth.ca/resources/finding-evidence/grey-matters
- https://www.science.gov/
- “NIH RePORTER”: https://projectreporter.nih.gov/reporter.cfm
- “The Grey Literature Report”: http://www.greylit.org/
- “OpenGrey”: https://www.opengrey.eu/
- “Mednar”: https://mednar.com/mednar/desktop/en/search.html
- “OSF registries”: https://osf.io/registries
- Eldis: https://www.eldis.org/
- R4D: http://www.r4d.ch/
- Agency for Healthcare Research and Quality: https://www.ahrq.gov/
- National Insitute for Health and Clinical Excellence: https://www.nice.org.uk

In addition to the electronic searches described above, we looked into websites of governmental health authorities, specific emergency outbreaks (eg, Ebola), used citation chasing and handsearching strategies for topical journals from 2014-2019. We also contacted experts and practitioners in the field for internal reports and unpublished literature. See Supplementary file 1 for a detailed search strategy, which has been adapted for other databases.

 As the aim of this synthesis was to better understand the key features of a resilient health system in low- and middle-income countries based on empirical evidence, the review only included applied cases where a major shock of political, environmental, and epidemiological nature had occurred. Primary studies were included if they used qualitative study designs such as case studies, ethnographies, and qualitative process evaluations, and studies that used both qualitative methods for data collection (eg, focus group discussions, individual interviews, observation, diaries, document analysis, etc) and data analysis (eg, thematic analysis, framework analysis, etc). Only studies published in English language were considered. Table 2 summarizes the inclusion and exclusion criteria.

**Table 2:** SPIDER Tool Analysis

**SPIDER**	**Inclusion **	**Exclusion**
S – Sample	Low- and middle-income countries as classified by the World Bank^a^	High-income countries as classified by the World Bank^a^
PI – Phenomenon of interest	Countries that underwent a major shock^b^ since 2014	Studies that contain no major shock^b^
D – Design	Case studies, ethnographies, qualitative process evaluations	Study design using only quantitative analysis
E – Evaluation	Lessons learnt, findings, perceptions, experiences relating with responses to an external shock that would unveil the capacity of health system resilience, building on the *a priori* conceptual framework for analysis^10^	N/A
R – Research type	Qualitative studies, mixed methods	Systematic reviews, quantitative-only studies

^a^ World Bank Group, 2019. https://datahelpdesk.worldbank.org/knowledgebase/articles/906519-world-bank-country-and-lending-groups. ^b^ A major shock here refers to an acute man-made or natural occurrence of crisis such as war, natural disaster, and new pandemics whether it be from an external or internal source. Shocks of political, environmental, and epidemiological nature were considered for our analysis.

**Figure 2:**
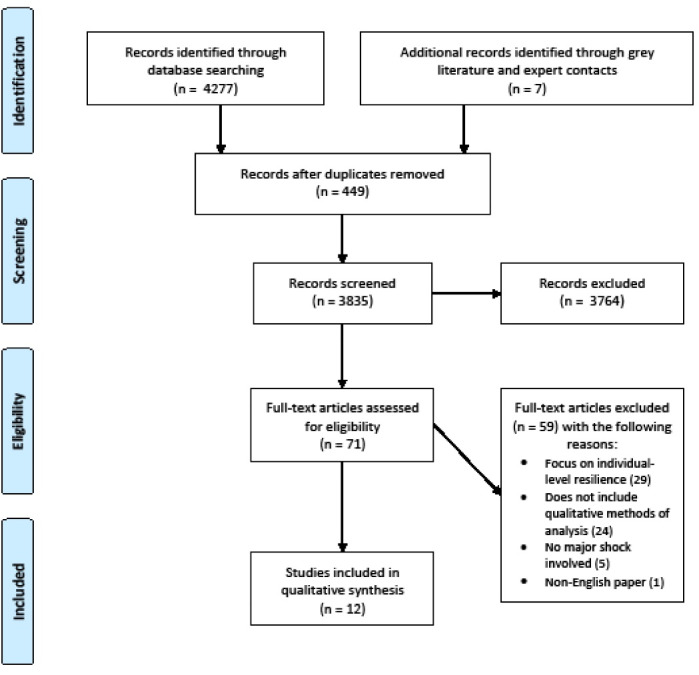
PRISMA Diagram of the Literature Search. Abbreviation: PRISMA, Preferred Reporting Items for Systematic Reviews and Meta-Analyses.

 First, study title and abstracts were screened for relevance and full-text articles were obtained for the second stage of screening for final eligibility. Detailed reasons for articles excluded were recorded and compared between authors to agree on discrepancies. Two authors independently reviewed all title/abstracts and full-text formats for the two-stage screening process. A systematic search identified 4284 unique citations from key electronic databases and reports by non-governmental organisations. Of the 4284 articles, 449 articles were discarded as duplicates, and an additional 3764 articles were excluded for irrelevance. Seventy-one full-text articles were assessed for eligibility, which resulted in a further 59 exclusions. Studies were excluded because they did not use qualitative methods, and the focus of resilience was not systemic but of individuals. Most of the grey literature reports did not meet the inclusion criteria due to compromised quality and the lack of depth in analysis. Twelve studies met the final inclusion criteria and were included in the synthesis (Figure 2).

###  Characteristics of Included Studies

Table 3 outlines the characteristics of the included studies. Overall, there was rich diversity in terms the geography (continent) and types of shock (environmental, political, epidemiological) covered in the papers. Four articles were from Asia, six from Africa, and two from the Middle East, representing a good contextual diversity. With regards to the nature of the shock, three were environmental, three were political, and six were epidemiological. As all studies were from low- and middle-income countries with comparable levels of health system, the findings were more likely to be transferrable and the common themes easily generated. All papers were of qualitative nature, discussed findings, experiences, perceptions, and lessons learnt responding to the shock, which revealed critical aspects of the capacity of resilience in its health system.

**Table 3:** Characteristics of Included Studies

**Author**	**Country**	**Study Design**	**Objective**	**Type of Shock**	**Study Timeline **
Achour et al, 2016	Turkey	Qualitative: interviews and focus group discussions	To explore how proactive approaches to natural hazards are operating on the ground	Environmental: earthquake	After the shock
Ager et al, 2015	Nigeria	Qualitative: interviews and workshop	To understand, predict and potentially influence the processes which support the resilience of health systems in contexts of adversity	Political: Boko Haram insurgency	During the shock
Alameddine et al, 2019	Lebanon, Jordan	Qualitative: key informant interviews	To advance the understanding of the resilience of health systems by interrogating the appropriateness of a capacity-oriented resilience framing via a case study of the UNRWA	Political: Syria crisis and the displacement of refugees	After the shock
Ammar et al, 2016	Lebanon	Case studies	To assess the resilience of the Lebanese health system in the face of an acute and severe crisis and in the context of political instability	Political: Syria crisis and the displacement of refugees	After the shock
Curran et al, 2018	Kenya	Mixed methods: health facility checklist, focus group discussions	To assess the cholera preparedness in several counties and explore clinic- and community-based healthcare workers experiences during the 2015 cholera outbreak response	Epidemiological/pandemic: cholera outbreak	During and after the shock
Farley et al, 2017	Sri Lanka	Mixed methods: questionnaire, in-depth interviews	To assess the flood preparedness in healthcare facilities in Eastern Province of Sri Lanka	Environmental: floods	After the shock
Lin et al, 2014	China	Mixed methods: literature review, key informant interviews	To describe and analyse the public health system response to the Sichuan province earthquake and to develop a conceptual framework that may be used by other researchers to describe and analyse the public health system response to other disasters	Environmental: earthquake	After the shock
Ling et al, 2017	Liberia	Qualitative: in-depth interviews and focus group discussions	To understand how a health system adapts to crisis and how the priorities of different health system actors influence this response	Epidemiological/Pandemic: Ebola outbreak	After the shock
McPake et al, 2015	Uganda, Sierra Leone	Case studies	To identify the factors that contributed to the relatively successful control of the Ebola outbreak in Northern Uganda compared to Sierra Leone and to trace the extent to which both outbreak and control were affected by the histories of the conflict in both settings	Epidemiological/Pandemic: Ebola outbreak	After the shock
Otu et al, 2018	Nigeria	Qualitative: key informant in-depth interviews	To describe the events that occurred during the Nigeria Ebola crisis in 2014 with a view to identifying successes and gaps in the containment response	Epidemiological/Pandemic: Ebola outbreak	After the shock
Purohit et al, 2017	India	Mixed methods: document review, in-depth interviews	To examine public health experiences in the 2009 H1N1 influenza pandemic in Pune, India	Epidemiological/Pandemic: H1N1 influenza pandemic	After the shock
Thiam et al, 2015	Guinea	Mixed methods: desk review, interviews, direct observations	To report the main challenges faced by the frontline health services and by the communities including their perceptions and views on the current Ebola response in the Prefectures of Coyah and Forecariah in Guinea	Epidemiological/Pandemic: Ebola outbreak	During and after the shock

Abbreviation: UNRWA, the United Nations Relief and Works Agency for Palestine Refugees in the Near East.

###  Data Extraction, Analysis, and Synthesis

 For screening and data extraction, EPPI-reviewer 4 was used to allow authors to collaborate on the same platform. A standard extraction tool was developed to extract relevant descriptive data and to code findings against the *a priori* coding framework. Basic data such as author, year of publication, study aim and objective, study type were extracted, as well as any relevant contextual information, description of the shock, and theoretical framework used. Themes outside of the *a priori *framework were collated to form a refined list of themes. Then, all twelve studies were revisited using the new list of themes to analyse them in the context of each study. One reviewer was responsible for the whole data extraction and a second reviewer reviewed 10% of a random sample of the included papers for quality assurance.

###  Quality Assessment

 The Critical Appraisal Skills Programme (CASP) checklist for qualitative research was adapted as a scoring system for the quality appraisal of included studies.^23^ Two authors independently appraised the quality of the papers considering the CASP scores and additional questions to assess the study quality by considering the study’s use of theoretical frameworks, robust analytical method, in-depth interviews and focus group discussions in the case of primary qualitative study. Each study was given an overall rating of outstanding, fair, and poor. After discussing discrepancies, the authors agreed to include all 12 studies regardless of the final rating, as studies that may not have ranked highly in the quality appraisal can still add value in corroborating the findings from higher quality studies with stronger explanatory power.

## Results

 Ten themes were generated from the analysis. Each of the theme was then compared with the elements of the health system that best corresponded to health system building blocks. We have distinguished ‘Health system elements’ as detailed components of the health system that manifested as strength or weakness of the given context when responding to crisis. The elements were grouped into the corresponding WHO health system building blocks.^22^ The first five were adapted from the *a priori *conceptual framework that characterizes a resilient health system: aware, diverse, self-regulating, integrated, adaptive.

###  Dynamic Attributes of Health System Resilience

 As described by Kruk et al, resilient health systems share key features that seem to apply across diverse contexts and nature of shocks.

####  Aware

 First, a resilient health system is aware of its system capacity and risks. Awareness is the capacity to detect and interpret local warning signs and quickly call for support.^11^ It can be measured through the knowledge and distribution of its health system assets, having an active epidemiological surveillance system, and an open communication channel that makes relevant information accessible through various forms of media.^11^

 In the aftermath of the 2011 Van earthquake, it was evident that Turkey’s rescue teams on the ground did not have sufficient information regarding the available resources and lacked the capacity to locate collapsed buildings. This led to a significant waste of time and resources for onsite inspections and heavy reliance on local sources that could have been spared with a systemic approach to surveillance. One interviewee noted that “it was difficult to find maps of the area to locate collapsed buildings, and gather information about the number of victims.”^24^ This indicates how a lack of awareness of its geo-registry of human resources, supplies and facilities during calm can cause a chain of inefficiencies when crisis hits.

 On the other hand, in Lebanon, during the Syrian refugee crisis that placed a considerable burden on its health system due to the unprecedented influx of refugees, the primary healthcare department, along with the epidemiological surveillance unit, played a crucial role in ensuring effective and ongoing surveillance of its disease burden.^25^ Key data of emerging infections and outbreaks were monitored and reported. Effective immunization coverage and the activation of its early warning and response system allowed for a successful prevention and control of communicable diseases such as measles, polio, cholera, and tuberculosis even during a crisis.^25^

 Awareness is associated with a strong health information system, as manifested in the included studies by a functioning surveillance and reporting system, as well as a transparent communication channel to the public (see Table 4).

**Table 4:** Relevant Health System Building Block for “Awareness”

**Health System Building Block**	**Information**	
Health system element	Transparent communication to the public and sensitization using the media	Functioning surveillance and reporting system
No. of studies shown as strengths	3	2
No. of studies shown as weakness	3	1

 The presence or lack of awareness were found to be critical in grasping and monitoring key information for the system to respond effectively in crisis situations.^20,24-30^ Having an independent epidemiological surveillance unit within the Health Ministry and regularly sharing the findings with other relevant ministries and the public not only served its purpose to deal with impending arising health needs but also had a stabilizing effect of gaining the trust of the partners and its people.

####  Diverse

 Second, a resilient health system responds to a diverse range of healthcare needs. Diversity here refers to the capacity to address a broad range of health challenges arising due to the crisis and meeting varying needs of its population.^10^ It can be measured through its scope of health services available in primary care, adequacy of government health expenditure and financial protection mechanisms.^11^

 Diversity was demonstrated in how the Chinese government handled the varying health needs after the devastating effects of the Sichuan earthquake in 2008. After dealing with the acute care stage of earthquake victims, treatment of non-earthquake related illnesses and chronic disease patients became the country’s next medical focus. “40% of the patients in Wenchuan county were over 60 years old….vulnerable population – such as older people, women and children – were highly represented, and the need for chronic healthcare was great.”^27^ Hospitals sent medical teams daily to temporary settlement areas to provide primary care services and health education for this population groups. In addition, three months after the earthquake, more than 40% of the earthquake survivors in the heavily affected counties suffered from post-traumatic stress disorder. China’s Ministry of Health quickly mobilised and dispatched experts and volunteers to offer mental health and social services, a targeted gesture to meet the impending needs of its population.^27^

 In contrast, Liberia’s Ebola crisis in 2015 revealed that in its early response, no health services were available because most health facilities were shut down.^20^ Even in later stages, the clinics that gradually opened offered limited health services. This was because prior to Ebola, health services were segregated into disease-based programmes according to international funding availability. The dependence on external support led to a highly verticalized health programming with patchy performance, giving no consideration to building a long-term healthcare system; for example, malaria was managed well whilst maternal health was not. The same pattern was repeated during Ebola, as Ebola treatment units that relied on external funding operated and health facilities used as triage stations to screen for Ebola symptoms, but most primary care facilities and hospitals were closed or offering limited services.^20^

 Diversity is associated with leveraging internal and external resources effectively to deliver a wide range of health services to its people, as exhibited by relevant studies (see Table 5).

**Table 5:** Relevant Health System Building Block for “Diversity”

**Health System Building Block**	**Service Delivery**	**Financing**
Health system element	Diverse range of health services offered to meet impending needs of target population groups	Leverage internal and external resources effectively
No. of studies shown as strengths	4	4
No. of studies shown as weakness	1	2

 The theme of diversity highlighted the importance of having a system-based approach to resilience, as countries require a strong foundation of health financing mechanisms, infrastructure, and human resource pool to flex its capacity when the diverse health needs arise.^20,24,25,27,31^ It was evident during the Ebola crisis that the reliance on short-term donor-based programmes diverted the country’s priority away from building a lasting health system.^20^

####  Self-regulating

 Third, a resilient health system is self-regulating and offers undisrupted health services to its people. Self-regulation is the ability to contain and isolate health threats while delivering core health services.^10^ It can be measured through its collaboration agreements with regional and global actors, especially non-state and private providers and a database of service delivery alternatives for affected and unaffected populations.^11^

 As soon as Ebola hit, health facilities were shut down in eastern and central Liberia, or they were severely understaffed, or barely functioning. Healthcare workers fled and there were limited county health team funds and capacity to respond to the crisis. As most primary care facilities and hospitals closed, deaths occurred as a result from the void of health services.^20^ In the case of Lebanon during the Syrian refugee crisis, however, the Lebanese health system sustained a level of financing services at all levels, primary, secondary, and tertiary. The Ministry of Health maintained and even improved contracting terms with private hospitals by including performance measures in the contracts to achieve required service volumes at specified quality levels. As a result, the provision of healthcare was sustained throughout the crisis and primary healthcare centres and hospitals from both public and private sectors have remained operational.^25^

 As demonstrated in all relevant studies, this stark difference in the capacity of self-regulation appear to be attributed to the level of leadership and governance upholding the health system.^20,25,27,31-33^ The ministry of health has taken the lead in these exemplary countries to set up collaborative agreements with a wide range of actors to ensure that the system runs and services extended to its people without disruption (Table 6).^25,27,31,32^

**Table 6:** Relevant Health System Building Block for “Self-regulation”

**Health System Building Block**	**Leadership and Governance**	**Service Delivery**
Health system element	Collaboration with a wide range of actors (other ministries, private, public, NGOs, etc)	Undisrupted services in times of crisis
No. of studies shown as strengths	8	2
No. of studies shown as weakness	3	1

Abbreviation: NGOs, non-governmental organisations.

####  Integrated

 Fourth, a resilient health system has a formal unified coordinating channel that is integrated and coordinates with non-health actors and engages with the community to respond to crisis. Integration is the capacity to bring together diverse actors, ideas, and groups to come up with solutions and initiate action.^10^ It can be measured through an existence of a national emergency coordination system, platforms for dialogue with community leaders, and various working groups and their roles and protocols.^11^

 Evidence of integration was apparent in multiple settings.^20,24-28,31-33^ Turkey took a proactive approach to merging many directorates to reduce bureaucracy and opt for a single approach for emergency response by having the Ministry of Health’s Disaster and Emergency Coordination Centre be in charge of the overall coordination in the disaster area.^24^ In the heat of the Boko Haram insurgency, the exceptional coordination between Nigeria’s State Ministry of Health and security forces enabled health workers to go to work during curfew hours.^32^ Critical in these coordination efforts were also the role of the community. Community members in Nigeria organized transportation access to health services and served as key sources of information, bridging the healthcare workers to the communities. They provided spiritual, emotional, and social support to each other to cope with the crisis together.^32^ In Liberia, however, a haphazard coordination between government actors and non-governmental organisations (NGOs), and the lack of transparency in budget management and Ebola surveillance data, exacerbated community suspicion. As a consequence, one NGO respondent described that this lack of coordination at every level led to a paralysis in actual progress and movement.^20^

 The capacity to integrate, therefore, comes from a strong foundation of leadership and governance and is also related with the government’s ability to communicate with its people in a transparent and consistent manner to gain the trust of its populace. Once the community engagement is strong, resilience in the health system is easily exhibited at all levels (Table 7).

**Table 7:** Relevant Health System Building Block for “Integration”

**Health System Building Block**	**Leadership and Governance**	**Other**
Health system element	Successful coordination structure created for the crisis	Strong community trust and engagement based on respect of culture
No. of studies shown as strengths	5	2
No. of studies shown as weakness	2	2

####  Adaptive

 Fifth, a resilient health system is adaptive to the evolving situation and deals with the crisis with flexibility. Adaptiveness is the ability to transform in ways that improve function in the face of highly adverse conditions.^10^ It can be measured through provisions to reallocate funds in emergency, management capacity of local health teams, agreement to delegate authority and funding in crises.^11^

 It was paramount that countries quickly shifted its *modus operandi* to the evolving situation. For instance, when strict curfew measures were introduced to restrict movement during the insurgencies, the Hospital Management Board in Nigeria called its senior physicians and nurses to make a formal change to the health worker duty shifts. Instead of three 8-hour shifts, two 12-hour shifts were established, which ensured continuity of staffing at facilities given its curfew restrictions.^32^ During the insecure period, medicine stock was redistributed to the facilities most affected by the influx of refugees in Lebanon. The United Nations Relief and Works Agency for Palestine Refugees in the Near East and health authorities in Lebanon bought 25% of the annual stock of medicine to prepare for emergency and ensure continuous availability of medicine.^31^ Evident in Nigeria’s account of the response to its Ebola outbreak, huge sums of money were made available to manage the outbreak and monetary incentives were provided by the Lagos State Government for medical volunteers to draw up life insurance policies and other incentives to reward those who work in the treatment centres.^28^ All these efforts are in line with leadership and governance especially at decentralized levels where resources can be shifted and allocated flexibly to meet the evolving situation and changing needs (Table 8).

**Table 8:** Relevant Health System Building Block for “Adaptive”

**Health System Building Block**	**Leadership and Governance**	**Financing**
Health system element	Successful decentralization of responsibilities	Fundraising and flexible allocation of resources to meet needs
No. of studies shown as strengths	3	4
No. of studies shown as weakness	3	2

###  Foundations of Resilience 

 Five new themes were generated from a thematic analysis of the data extracted from all the included studies. These themes did not fit neatly into the *a priori* framework, but came up as reoccurring themes that revealed a pattern in itself. These new themes were realigned relationships, foresight and motivation, change management, and emergency preparedness. Based on their functions, these five new themes were re-grouped into two sub-categories: drivers of resilience and organisational mechanisms. The drivers of resilience were features of resilience that propelled the health system to overcome structural barriers, whilst the organisational mechanisms were the enabling structures of the health system that activated resilience. These two sub-categories complement one another, as the dimensions of software and hardware are both required in building the resilience of the health system.

####  Drivers of Resilience: Realigned Relationships, Foresight, Motivation 

 First, a resilient health system responds to disruption by realigning working relationships and building mutual trust between countries, teams and individuals to work collaboratively towards shared goals.

 Despite diplomatic controversies between neighbouring countries, China sought relief experts and rescue operation equipment soon after the Sichuan earthquake. China even accepted support from at least three private relief teams in Taiwan, with whom China had a long tense relations.^27^ This was an exemplary decision that showed a new working relationship overcoming historical and cultural boundaries in times of crisis under a shared goal to save lives. Likewise, St. Mary Lacor hospital in Gulu, a well-equipped, faith-based, NGO hospital filled the gap in the health sector in Northern Uganda. The Ebola outbreak placed a significant pressure in its health system that had been affected by two decades of conflict. It offered a consistent and high standard of care, and created a safe space for people fleeing violence.^33^ In the cases of China and Uganda, external partners played a positive role, as there were trust and openness that overcame barriers to work towards the same vision.

 This was not the case in Ebola-stricken Liberia. The Ministry of Health and NGOs were hesitant to disclose funding and Ebola surveillance data, which led to community suspicion and paralysis in relief efforts.^20^ In Guinea during the Ebola crisis, the main challenges encountered by the frontline health workers and communities stemmed from the lack of trust and shared goals. “The coordinators have been parachuted from the top, without asking for our opinion,” a health personnel from one prefecture attested. There were strong resistance from the communities to Ebola control interventions because of the lack of proper sensitization measures for safe burial practices. The use of foreigners instead of locals in raising awareness further eroded the trust and was seen as an invasion.^30^ It was clear that the human components, in this case, the respect of local culture and engagement of the communities, contributed to building trust, which were foundational in achieving resilience of its system.

 Second, a resilient health system has foresight to invest in long-term slow variables of the health system infrastructure and human resources.

 One of the common patterns of failed approaches to responding to a crisis was to prioritize on the so-called ‘fast variables.’ This was most evident in Liberia during the Ebola crisis, as donors and NGO funding focused heavily on delivering these ‘fast variables’ in the form of temporary infrastructures, surveillance teams, infection prevention supplies, and isolation units that reached Liberia within months, whilst disregarding a longer term health system resilience. There were huge investments towards health worker training on infection prevention, but no emphasis on building a cadre of well-trained nurses that can serve the health system as a whole.^20^ Similar problems plagued Sierra Leone and Nigeria during the Ebola crisis, as the health workforce was chronically understaffed, demotivated, and ill-equipped. These factors exacerbated the growing mistrust between service providers and communities.^33^ In Nigeria, a medical doctor reported that “a big challenge was a lack of standard quarantine station. The decision to use hospital Y as a treatment centre was a late one.” Likewise, a lack of long-term infrastructure was reported to be one of the main challenges faced.^28^

 Meanwhile, in Lebanon, deliberate Ministry of Health policies to establish a career path for nurses, investing in training more nurses, and increasing nurses’ salaries all contributed to a steady increase in the number of nurses working in the Lebanese health system even during the Syrian crisis. The health workforce catered to both refugee and Lebanese populations in challenging times.^25^ Turkey also invested heavily in improving the structural capacity of its infrastructure by launching the EUR 1.129 million Istanbul Seismic Risk Mitigation and Emergency Preparedness project. A significant reform work for healthcare infrastructure renovation was undertaken to construct new healthcare facilities, and renovating existing facilities to mitigate seismic risk that are resistant to earthquakes.^24^ These were all efforts to ensure long-term infrastructural readiness before the crisis. Building resilience is much more than preparedness, as echoed by Kruk et al, that it is about investing in systems and other ‘slow variables’ that can build a strong health system that functions both in crisis and in calm.^11^

 Third, a resilient health system shows a high level of motivation, exhibited by strong political will and personal commitment.

 A strong sense of political will from the government had a stabilizing effect for frontline workers and the public. In Nigeria, the State Governor paid multiple visits to the hospitals and other facilities in the aftermath of major insurgencies and took active measures to improve infrastructural conditions, drug supplies, and human resource capabilities.^32^ Chinese President Hu Jintao ordered a rapid disaster response effort to help the victims and communicated to reassure the public minutes after the earthquake. Premier Wen Jiabao also flew to the area within 90 minutes after the earthquake to manage rescue efforts. Interviewees reported a positive influence of these decisive leadership and political will. “His presence on the scene had a positive impact on subsequent decisions, as the premier made such decisions with direct knowledge of the situation.”^27^

 In Kenya, cholera outbreak responses were exemplary, which stemmed from committed healthcare workers willing to work overtime despite resource limitations. One health worker noted “if it were not the passion, the spread would have gone up, because without funds you have to have the passion to work on it.” A deep personal commitment of frontline workers held the health system together no matter how fragile and resource constrained the context seemed. Often these healthcare workers felt a sense of achievement and reward when they overcame resource challenges and showed a strong coordination between partners at the clinic and community levels.^26^

 These first three themes namely, realigned relationships, foresight, and motivation, were shown to be the key intangible ‘drivers of resilience’ that enables the system to cope with shocks even in resource-constrained settings. These themes also relate with the health system building blocks that require a permanent and long-term investment (Table 9).

**Table 9:** Relevant Health System Building Blocks for the “Drivers of Resilience”

**Health System Building Block**	**Service Delivery; Medical Products, Vaccines and Technologies **	**Health Workforce**		**Other**
Health system element	Long-term infrastructural readiness	Adequate supply of trained health workforce	Staff motivation and commitment	Strong community trust and engagement based on respect of culture
No. of studies shown as strengths	3	3	4	2
No. of studies shown as weakness	5	4	0	2

####  Organisational Mechanisms: Change Management and Emergency Preparedness

 Fourth, a resilient health system has a well-integrated system of new initiatives and reforms.

 There was a remarkable difference between the Jordanian and Lebanese systems during the immediate crisis periods, relating to the newly introduced health system reforms, the Family Health Team model and the e-health system. In Lebanon, both reforms had only recently been introduced and placed a significant burden on its staff to fill both paper and electronic records. In contrast, these reforms had been implemented relatively early in Jordan. One of the support staff mentioned that staff got relieved with the introduction of the e-health and the appointment function was helpful in managing high flow of patients which came with the crisis.^31^ New initiatives and reforms on health system can only build resilience when well-integrated into the system.

 On the contrary, recent major changes to the health system governance in Kenya, specifically the decentralization of health services, may have contributed to the confusion of roles, reporting, and supply chain management. Some healthcare workers believed that the confusion of roles and responsibilities during the cholera response was due to the transition from a national health system to a decentralized system managed by county governments.^26^ A study in Kilifi, Kenya identified a similar health system challenge, where the system’s devolution seemed to aggravate issues.^34^ Changes in the health system structures and initiatives that are not fully integrated into the national health system appeared to have exacerbated challenges experienced from the crisis.

 Fifth, a resilient health system has an emergency preparedness strategy in its national health system and implements it at all levels.

 The Ministry of Health in Lebanon called upon international agencies to consider a strategy to integrate planning, financing, and service delivery by embedding refugee healthcare within the national health system. Through an establishment of a steering committee, a “Lebanese crisis response plan” was drafted outlining all funding sources, activities, and coordination efforts. This integrated approach to refugee healthcare made it possible for the refugees to settle and access the same level of healthcare in Lebanon rather than to have a parallel system of delivery and financing systems care that are concentrated in the camps.^25^

 Having a strategy is however only the first step. Turkey had a four-tier triage protocol in times of the earthquake: ambulatory, delayed, immediate, and deceased. This protocol, however, was not always used during the evacuation and dispatch processes. An interviewee stated that “everybody knows what triage is, but they don’t use it.” Having a policy and strategy in place did not automatically translate into practice. As evident in Turkey’s case, the triage protocol which was excellent on paper turned out to be futile during the emergency.^24^ When evaluating flood preparedness in Sri Lanka, only around 13% of the health facilities had an emergency plan, all of which were in writing. Most healthcare workers never received trainings nor had received any instructive documents on disaster preparedness from the central government.^35^ The frustrations of the lack of systematic planning were echoed from hospital officials in India as well. “We Indians are better at dealing with a crisis situation, meaning we can tackle the crisis very well but we don’t have a long term planning. Though the experience has increased awareness about influenza preparedness. Still we do not have public health machinery to document the influenza burden.”^36^

 These additional two themes which focused on change management and emergency preparedness served as ‘operational mechanisms’ for the system to be equipped with preparedness strategies that can be translated into action when required. These themes relate with leadership and governance of the health system to establish laws, policies, and strategies and have the mechanism to enforce them at all levels (Table 10).

**Table 10:** Relevant Health System Building Blocks for the “Organisational Mechanisms of Resilience”

**Health System Building Block**	**Leadership and Governance **	
Health system element	Enabling law, policy and strategy and enforcement	Health system reforms well integrated and established
No. of studies shown as strengths	7	2
No. of studies shown as weakness	1	2

## Discussion

**Figure 3:**
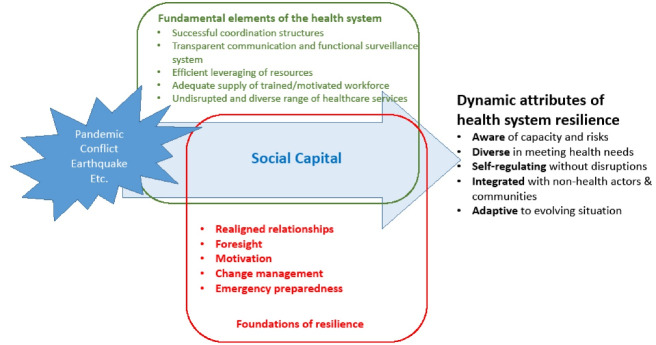
Refined Conceptual Model of Health System Resilience.

 Ten themes generated from this review were mapped onto a refined conceptual model as presented in Figure 3. This figure tracks conceptually the journey from health system shock to the five themes indicative of resilient systems from Kruk and colleagues’ original framework,^11^ through five new themes that were attributes leading to resilience and elements of health systems that were strengths and weaknesses when responding to crises. The influential elements of health systems and the five attributes leading to resilience shared a dependence on social capital which was central to driving resilient responses. As health systems are complex and evolving in nature, it is vital that resilience is viewed as a capacity that can be built rather than a static attribute, which underpins this review’s findings.^37^ Similarly, there is now a call to conceptualise health system strengthening, not just health system structures.^38^ Common to both health system strengthening and resilient responses to crises is social capital, which is comprised of relationships, trust, shared goals and collaboration. Social capital is pivotal in both crisis and calm, and critical in building the health system as well as to enhance resilience.

 Blanchet et al used systems thinking and complexity theories to frame the governance of resilience as a capacity to anticipate and respond to uncertainties across a complex system, drawing on different forms of knowledge supported by the legitimacy of trusted institutions and widely held norms.^13^ Biddle et al applied this framework to the empirical literature and found that studies typically addressed single components of health systems rather than an inter-connected system as a whole and found a marked discrepancy between the frameworks of resilience and the applied cases of resilience, discrediting the legitimacy of the framework.^14^

 By using a best-fit framework synthesis, this review employed Kruk colleagues’ resilience framework, which was built upon the experiences from the Ebola response and frameworks from other disciplines.^10,11^ Not only did the review confirm the applicability of the framework’s themes, but also revealed additional themes in the literature. These empirical findings confirm the role of trust and institutional legitimacy proposed by Blanchet et al and add to Biddle and colleagues’ empirical analysis by identifying social capital as a key interconnecting mechanism to unlock the resilience of a health system.

 There are several additional observations that are relevant in explaining the mechanics of resilience.

 First, when there is an acute shock, the health system has to consider the numerous challenges that are amplified by its unique contextual vulnerabilities. This was evidenced in Nigeria where insecurity and movement restrictions from the insurgencies bred fear and anxiety in people that led to a mass migration of health workers, exposing the already fragile human resource for health conditions.^32^ Likewise, an abrupt transition from a national to a decentralized system in Kenya led to major confusion in roles and responsibilities that resulted in inefficiencies in the cholera response efforts due to duplicated roles and coordination failures.^26^ Fridell and colleagues’ recent scoping review underscores the importance of adjusting to long-term changes in the health system as a key feature of resilience which reflects the country’s leadership and governance.^39^ Hence, health systems should first focus on building its ‘everyday resilience,’ to deal with the day-to-day, chronic struggles arising from its weak governance and political and economic instabilities that are inherent in the system, in order to better prepare for future shocks.^12,40^

 Second, the foundations of resilience appear to be critical especially in resource-constrained settings to unlock the dynamic attributes of resilience suggested by Kruk et al^11^ in order for the system to achieve resilience. This requires a systems thinking approach to redefine the health system to serve its evolving purpose.^41^ Whether it be health systems of institutions or countries, taking a purpose-driven approach compels a transformation in its leadership, communication strategies, infrastructure, stakeholder engagement, and data surveillance methods.^42^ This interplay between the foundations and the dynamic attributes of resilience extends Abimbola and Topp’s definition of resilience as ‘adaptation with robustness.’ The authors argue that adaptation without robustness results in coping, whereas adaptation in the context of robustness brings resilience.^43^ Robustness here refers to the capacity of the system to recover from shocks.^43^ It is the designing and redesigning of the country’s institutions in anticipation of future shocks, which is precisely the building of long-term foundations resilience with a purpose-driven approach. The criticism that came from India’s hospital officials regarding the tendency of Indian public health services to ignore planning needs until a crisis demanded a response is the evidence of ‘coping’ as a result of the lack of foresight required to quickly shift the health system’s purpose to deliver health services in crisis.^36^ A health system with a clear vision in serving its people in both crisis and calm will duly strengthen its health system infrastructure and train its human resource cadre to build the foundations of its health system. Only then, can the system exhibit the dynamic features of resilience, being ‘aware’ of its capacity and risks, meeting the ‘diverse’ needs, and ‘adapting’ to the evolving situation.

 Third, strong working relationships, often realigned, provided a glue to the health system that enabled low- and middle-income countries to overcome resource constraints and structural barriers. This shared sense of vision to deliver health services to crisis-affected population enabled two historically tense nations to overcome political barriers and a faith-based NGO hospital to fill the void of the public healthcare system.^27,33^ This notion is echoed by Barasa and colleagues’ systems intangible ‘software’ (eg, norms, power, trust), which was identified as the key driver of resilience that can activate the attributes of system resilience.^44^ Topp stresses that health systems are social systems manifesting complex power relations, and that resilience is highly dependent on the way the health system is governed.^37^ Blanchet echoes the importance of developing legitimate institutions that can enable systemic resilience.^13^ The cases of highly resilient health systems in this review exhibited ‘realigned relationships’ as a response to shock management, which indicates that the governance structure of the health system was temporarily shifted in favour of the common goal. Existing bureaucratic procedures, hierarchies, decision-making protocols were at times bent in order to work on collaborative terms.

 Finally, this review has confirmed that the facets of resilience are not only reserved for strong health systems in high-income countries, but are capacities that can be acquired through a process of reflection, learning and devising creative ways of building the foundations of resilience. The review’s inclusion of only low- and middle-income countries highlights the key features of resilience regardless of the status of its health system. Van des Pas’ view of resilience as a moral obligation for all countries implies that building resilience is an investment towards extending global public goods for health.^17^

## Strengths and Limitations

 This review is the first to apply a best-fit framework synthesis approach to identify key features of resilience using empirical evidence. This allowed us not only to test Kruk and colleagues’ framework with empirical data, but to build on it through further empirical analysis. It also has the potential to make a timely contribution to the global health systems literature based on its refined conceptual model that can be applied to low- and middle-income contexts. It adopts systematic methods and internationally recognised reporting standards. As the review commenced before the COVID-19 outbreak, however, studies from the latest pandemic has not been included. The authors were still able to draw upon their immediate experiences during the pandemic in different contexts to recognise gaps in the evidence. Among these were the lack of attention given to social determinants of health, health inequalities, and the role of party politics, all critical aspects revealed from the COVID-19 crisis that affect resilience. Further studies should explore elaborating on these topics and for these concepts to be substantiated with real world evidence, and to be tested for their applicability in high-income countries as well.

## Conclusion

 To date, this is the first review to test the attributes of resilience specifically in the health sector and which offers a refined framework that can be applied in low- and middle-income settings. This review prompts low- and middle-income countries to consider building the foundations of resilience described here as a priority to better prepare for future shocks. These foundations for achieving resilience are realigned relationships, foresight, motivation, emergency preparedness, and change management. A collective response on the topic is of prime importance for all countries in order to adhere to the International Health Regulations and its global health security agenda if we are to minimize the damage of the next pandemic.

## Ethical issues

 Not applicable.

## Competing interests

 Authors declare that they have no competing interests.

## Authors’ contributions

 PYG, SO, SM, and KW conceptualized the review; PYG and WWH conducted the literature search, selection and data extraction; PYG and SO conducted the analysis and synthesis; PYG developed the first draft of the paper. All authors contributed to subsequent and final drafts.

## Disclaimer

 The views expressed in the submitted review article are of the authors’ and not representing an official position of the institution nor the funding source.

## Funding

 This article is part of the PhD project that has received funding from the European Union’s Horizon 2020 research and innovation programme under the Marie Skłodowska-Curie grant agreement No 801076, through the SSPH*+ Global PhD Fellowship Programme in Public Health Sciences (GlobalP3HS) of the Swiss School of Public Health*. The corresponding author is also a recipient of the Swiss Government Excellence Scholarship (ESKAS), provided by the Swiss Federal Commission for Scholarships for Foreign Students (FCS). The funding bodies did not play a role in the design, analysis, and writing of the manuscript.

## Supplementary files

Supplementary file 1. Search Strategy (Ovid MEDLINE).
